# Supplementary material for: An Ant-Mimicking Jumping Spider Achieves Higher Predation Probability with Lower Success Rate When Exposed to Ethanol
Source: Insects. 2022 Nov 1;13(11):1009. doi: 10.3390/insects13111009 (PMC9694002; doi:10.3390/insects13111009)
Supplement: Supplementary file 1 [file insects-13-01009-s001.zip › insects-1973150-supplementary.pdf]

An ant-mimicking jumping spider achieves higher predation probability with lower success rate when exposed to ethanol

Guocheng Yu [a,b], Zichang Li [a,b], Yao Zhao[b], Jie Liu[a], Yu Peng[a,*]

Supplementary Tables

Table S1. The *PI* (preference index) values and the results from a one-sample *t*-test for significant differences in *PI* with 0 of *Myrmarachne gisti*.

Concentration (%)	PI (Mean ± SE)	t	df	*P*
20	0.17 ± 0.08	2.063	59	0.044
25	0.17 ± 0.06	2.712	59	0.009
30	0.20 ± 0.07	3.061	59	0.003
45	-0.36 ± 0.07	-5.166	59	<0.001
50	-0.39 ± 0.07	-5.177	59	<0.001
100	-0.42 ± 0.07	-5.920	59	<0.001

Table S2. The *PI* (preference index) values and the results from a one-sample *t*-test for significant differences in *PI* with 0 of *Myrmarachne gisti* females.

Concentration (%)	PI (Mean ± SE)	t	df	*P*
20	0.25 ± 0.10	2.455	29	0.020
25	0.25 ± 0.07	3.558	29	0.001
30	0.19 ± 0.09	2.052	29	0.049
45	-0.41 ± 0.10	-4.008	29	<0.001
50	-0.29 ± 0.10	-2.807	29	0.009
100	-0.37 ± 0.11	-3.221	29	0.003

Table S3. The *PI* (preference index) values and the results from a one-sample *t*-test for significant differences in *PI* with 0 of *Myrmarachne gisti* males.

Concentration (%)	PI (Mean ± SE)	t	df	*P*
30	0.21 ± 0.09	2.230	29	0.034
35	0.25 ± 0.12	2.070	29	0.047
45	-0.32 ± 0.10	-2.649	29	0.013
50	-0.48 ± 0.11	-4.627	29	<0.001
100	-0.46 ± 0.08	-5.679	29	<0.001
